# Comparative risks of mortality, cardiovascular events, and dementia with methotrexate versus hydroxychloroquine in rheumatoid arthritis: a retrospective cohort study

**DOI:** 10.1097/MS9.0000000000004182

**Published:** 2025-10-28

**Authors:** Bal Krishna Subedi, Naveen Gautam, Rafal Ali, Anuja Upadhyay, Prakriti Sapkota, Basu Dev Subedi

**Affiliations:** a Department of Internal Medicine, Jefferson Einstein Montgomery Hospital (JEMH), East Norriton, PA, USA; b Department of General Medicine, Gulmi Durbar Basic Hospital, Gulmi, Nepal; c Department of Rheumatology, Jefferson Einstein Philadelphia Hospital, Philadelphia, PA, USA; d Department of Internal Medicine, Vassar Brothers Medical Center, Nuvance Health, Poughkeepsie, NY, USA

**Keywords:** all-cause mortality, end-stage renal disease, hydroxychloroquine, interstitial lung disease, methotrexate, NSTEMI, pulmonary hypertension, retrospective cohort study, rheumatoid arthritis

## Abstract

**Background:**

Rheumatoid arthritis (RA) is a chronic autoimmune disorder primarily affecting joints. Methotrexate (MTX) and hydroxychloroquine (HCQ) are common disease-modifying antirheumatic drugs, yet more comparative outcome studies are needed. This study aimed to compare associations of HCQ versus MTX initiation with mortality, cardiovascular events, and other major health outcomes in RA patients. This retrospective cohort study utilized the TriNetX database (electronic health records from 142 global health care organizations) to compare selected 10-year outcomes in RA patients starting HCQ or MTX therapy.

**Methods:**

Patients aged 18–75 years diagnosed with RA (ICD-10 codes) were studied. This retrospective, multi-center cohort study used data from the TriNetX Research Network (analysis: 23 March 2025). Two cohorts, HCQ initiators and MTX initiators, were created. Propensity score matching (PSM) balanced cohorts for age, sex, race, pre-specified comorbidities, and concomitant medications. Primary outcomes were 10-year cumulative incidences of all-cause mortality, non-ST elevation myocardial infarction (NSTEMI), ST-elevation myocardial infarction (STEMI), pulmonary hypertension (PAH), end-stage renal disease (ESRD), interstitial lung disease (ILD), osteoporosis, and Alzheimer’s dementia.

**Results:**

After PSM, 56 362 patients were in each cohort. HCQ initiation was associated with significantly higher 10-year relative risks (RR) for NSTEMI (RR: 1.217), ESRD (RR: 2.624), ILD (RR: 1.377), PAH (RR: 1.625), and all-cause mortality (RR: 1.379) compared to MTX. No statistically significant RR differences were observed for Alzheimer’s dementia, STEMI, or osteoporosis.

**Conclusion:**

In this large, real-world observational study, RA patients initiating HCQ had a higher 10-year risk association for ESRD, ILD, PAH, NSTEMI, and all-cause mortality versus MTX initiators. These findings highlight potential differences in systemic risk profiles and are hypothesis generating. However, the results must be interpreted with significant caution due to major limitations, including the potential for residual confounding from unmeasured variables like disease severity and the absence of adherence data. Further prospective research that addresses these limitations is warranted to confirm these associations and explore underlying mechanisms.

## Introduction

Rheumatoid arthritis (RA) is a chronic, systemic autoimmune disease primarily characterized by synovial joint inflammation, leading to progressive joint damage, functional disability, and reduced quality of life. Beyond its articular manifestations, RA carries a significant systemic inflammatory burden, increasing the risk for extra-articular comorbidities like cardiovascular disease, interstitial lung disease (ILD), and kidney disease, ultimately impacting morbidity and mortality^[1,2]^. Consequently, RA management aims to achieve sustained remission or low disease activity, prevent structural damage, and mitigate these systemic consequences^[3,4]^. Disease-modifying antirheumatic drugs (DMARDs) are central to therapy, with conventional synthetic DMARDs (csDMARDs) often forming the foundation of treatment strategies^[3,4]^.

Among csDMARDs, methotrexate (MTX) is the established anchor therapy for moderate-to-high disease activity due to its efficacy in controlling inflammation^[3,4]^. Hydroxychloroquine (HCQ), another common csDMARD, is often used for milder RA or in combination therapy^[1,3]^. While generally considered less potent than MTX monotherapy for joint inflammation, HCQ has potential extra-articular benefits, such as improved lipid profiles, though risks like retinopathy require monitoring^[1]^. Despite their widespread use, large-scale comparisons focusing on the long-term effects of MTX versus HCQ initiation on major systemic comorbidities and mortality in real-world settings remain limited, representing a critical knowledge gap given RA’s systemic nature and the drugs’ distinct profiles.

Real-world evidence (RWE), derived from analysing real-world data (RWD) like electronic health records (EHRs), provides a valuable means to investigate long-term outcomes in diverse patient populations, complementing data from randomized controlled trials^[5]^. Utilizing platforms such as TriNetX, which aggregates global de-identified patient data, allows for such large-scale RWE analyses. Therefore, this study leveraged the TriNetX platform to compare the long-term risk of developing specific major comorbidities – Alzheimer’s dementia, non-ST elevation myocardial infarction (NSTEMI), ST-elevation myocardial infarction (STEMI), pulmonary hypertension (PAH), end-stage renal disease (ESRD), and ILD – as well as all-cause mortality, between patients with RA initiating treatment with either MTX or HCQ. The primary objective was to assess these comparative risks after attempting to balance baseline patient characteristics through propensity score matching (PSM). This cohort study has been reported in line with the STROCSS 2025 guidelines^[6]^.

## Methods

### Study design and data source:

This investigation was a retrospective, multi-center cohort study conducted using de-identified patient-level data from the TriNetX Research Network (“Global Collaborative Network”). The TriNetX platform aggregates EHR data from a diverse network of health care organizations (HCOs). At the time of analysis, the network included 142 HCOs, primarily located in the United States. The study was based on an analysis generated by the TriNetX platform on 23 March 2025, and there were no major deviations from the initial study design plan or timeline during the research. The study complied with TriNetX data use agreements and relevant privacy regulations. Each participating HCO is responsible for its own Institutional Review Board or ethics committee approvals for data contribution to the de-identified database. Given the secondary analysis of pre-existing, fully de-identified data, specific patient consent for this analysis was not required. Data quality within the TriNetX network is supported using standardized terminologies (e.g., International Classification of Diseases, 10th Revision, Clinical Modification [ICD-10-CM] for diagnoses, NLM:RXNORM for medications) and data curation processes maintained by the platform and contributing HCOs. This retrospective cohort study was registered in the Research Registry (unique identifying number: researchregistry11215) prior to data analysis. The study was conducted based on the methods outlined in the registered protocol and described herein.

### Patient cohort identification and index event:

Two primary cohorts were constructed using data from the TriNetX network. Cohort 1 (RA on HCQ) included patients aged 18–75 years with a diagnosis of RA (ICD-10-CM: M06.9) who initiated HCQ (NLM:RXNORM:5521) and did not have evidence of MTX (NLM:RXNORM:6851) use at cohort entry. Cohort 2 (RA on MTX) included patients aged 18–75 years with an RA diagnosis (ICD-10-CM: M06.9) who initiated MTX (NLM:RXNORM:6851) and did not have evidence of HCQ (NLM:RXNORM:5521) use at cohort entry. The ICD-10-CM code M06.9 (RA, unspecified) was chosen to maximize the cohort size by including a broad population of patients with an RA diagnosis, as it is a commonly used code in clinical practice, particularly when more specific classifications (e.g., seropositivity) are not immediately documented. This approach, however, may introduce some heterogeneity into the study population. The initial number of patients meeting these criteria before any analytical exclusions or matching were 70 388 for the HCQ cohort and 77 835 for the MTX cohort.

**HIGHLIGHTS**

- While methotrexate (MTX) and hydroxychloroquine (HCQ) are common treatments for rheumatoid arthritis (RA), more extensive comparative data on their long-term systemic risks are needed.
- This large, retrospective cohort study utilized propensity score-matched (PSM) real-world data from the TriNetX network (56 362 RA patients per group) to compare the 10-year risks of mortality, cardiovascular events, and other major health outcomes following initiation of HCQ versus MTX.
- After PSM, HCQ initiation was associated with a significantly higher 10-year relative risk of all-cause mortality, non-ST elevation myocardial infarction, end-stage renal disease, interstitial lung disease, and pulmonary hypertension compared to MTX initiation.
- No statistically significant differences in 10-year risk were observed between the HCQ and MTX groups for Alzheimer’s dementia, ST-elevation myocardial infarction (STEMI), or osteoporosis.
- In this real-world setting, RA patients initiating HCQ showed associations with higher long-term risks for several critical systemic outcomes compared to those starting MTX. These findings are hypothesis generating and must be interpreted cautiously due to significant study limitations, underscoring the need for further research to confirm these findings.

The identification of these cohorts from the database did not encounter significant challenges beyond the standard complexities of defining specific clinical criteria using EHR data. The index event for each patient was defined as the date of the first qualifying medication initiation (HCQ for Cohort 1 and MTX for Cohort 2) occurring on or after the RA diagnosis date. To be included in the analysis, the index event must have occurred within 20 years prior to the analysis date of 23 March 2025 (i.e., on or after 23 March 2005). Information on specific formulations, exact dosages beyond initiation, routes of administration, or treatment durations for the index drugs was not available for this analysis, which is a common limitation of studies using large RWD sources focusing on treatment initiation. Outcomes were assessed over a 10-year (3650-day) period, starting 1 day after the index event. For each specific outcome analysis, patients with a diagnosis of that particular outcome prior to or on the start date of the 10-year analysis time window were excluded from that specific analysis to ensure an incident cohort.

### Sample size and PSM

The sample size for this study was determined by the number of eligible patients available within the TriNetX database meeting the defined inclusion and exclusion criteria for the specified study period. Given the large scale of the database and the retrospective, hypothesis-generating nature of this research, a formal a priori sample size calculation for statistical power was not performed; instead, the study aimed to leverage all available data to maximize precision and generalizability.

To reduce potential confounding by indication, 1:1 PSM was performed. The cohorts considered for PSM comprised 65 669 patients in the HCQ group and 71 560 in the MTX group. Propensity scores, representing the probability of initiating HCQ versus MTX, were estimated using a multivariable logistic regression model. This model included several baseline characteristics extracted at or before the index date. Demographics included Age at Index, Sex (Female, Male), and Race (White, Black, or African American, Hispanic, or Latino). Diagnoses (Comorbidities) incorporated were Other hypothyroidism (ICD-10-CM: E03), Diabetes mellitus (E08-E13), Gout (M10), Chronic kidney disease (N18), Cerebrovascular diseases (I60-I69), Hypertensive diseases (I10-I1A), Hyperlipidemia, unspecified (E78.5), Overweight and obesity (E66), Tobacco use (Z72.0), and Other specified injuries of head, initial encounter (S09.8XXA). Concomitant medications included were aspirin, atorvastatin, adalimumab, etanercept, golimumab, infliximab, certolizumab pegol, sulfasalazine, tocilizumab, abatacept, tofacitinib, upadacitinib, leflunomide, metoprolol, rosuvastatin, simvastatin, and metformin. Patients with missing data for covariates included in the PSM model were excluded from the matching process by the algorithm, ensuring that matching was performed on records with complete information for these variables. After PSM, 56 362 patients remained in each cohort. Balance of baseline covariates after matching was assessed using Standardized Mean Differences (SMDs), with an SMD < 0.1 generally considered to indicate negligible imbalance.

### Outcomes of interest

The primary outcomes of interest were the 10-year cumulative incidences of all-cause mortality (identified by “Deceased” status in the database, which represents death from any cause and does not differentiate between specific causes such as accidents, cancer, or infections); NSTEMI (ICD-10-CM: I21.4); STEMI (ICD-10-CM: I21.3); PAH (ICD-10-CM: I27.0, I27.2); ESRD (ICD-10-CM: N18.6); ILD (ICD-10-CM: J84.9); Osteoporosis (with or without current pathological fracture; ICD-10-CM: M81.0, M80); and Alzheimer’s dementia (identified by Alzheimer’s disease; ICD-10-CM: G30).

### Statistical analysis:

The primary measure of association was the relative risk (RR) of experiencing each outcome within the 10-year follow-up period. For each outcome, the cumulative incidence (risk), risk difference, RR, and odds ratio, along with their 95% confidence intervals (CIs), were calculated for the matched cohorts using the TriNetX platform’s “Measure of Association Analysis.” The cumulative incidence (risk) was calculated as the number of patients experiencing an event divided by the total number of patients in the cohort. *P*-values for the comparison of risks were derived from a *z*-test for the significance of the risk difference. A two-sided *P*-value < 0.05 was considered statistically significant for the primary analyses. No pre-specified subgroup analyses were planned, and no sensitivity analyses (e.g., inverse probability weighting) were performed. Furthermore, no adjustments were made for multiple comparisons (e.g., Bonferroni correction), which increases the probability of Type I errors. All analyses were performed using the analytical tools integrated within the TriNetX platform.

## Results

**Figure 1.**
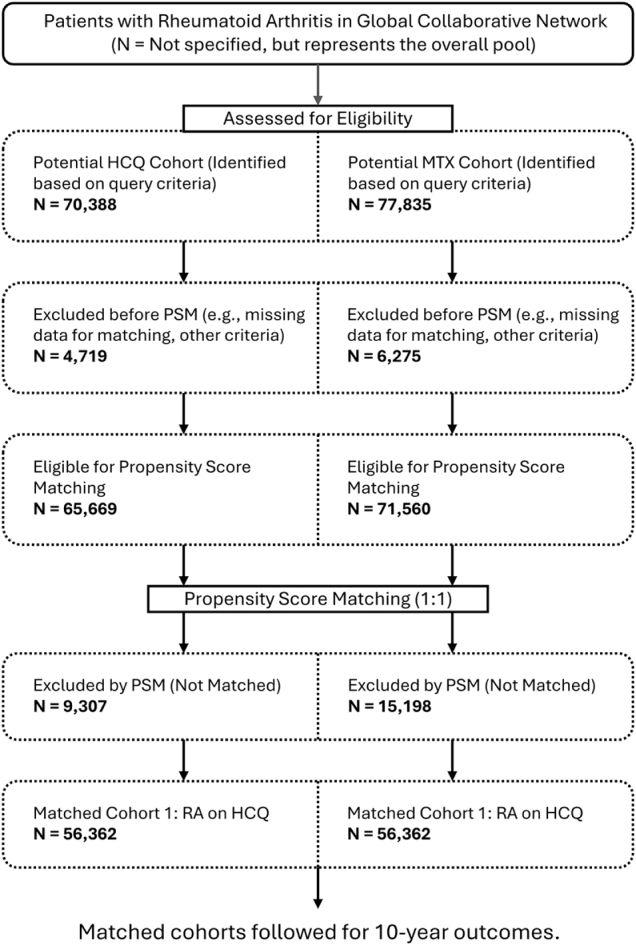
Flow diagram of patient selection for the retrospective cohort study.

The flow of patient selection through the study is detailed in Fig. 1. Following PSM, two cohorts of 56 362 RA patients each, who initiated treatment with either HCQ or MTX, were established. Baseline characteristics, including demographics, comorbidities, and concomitant medications, were comparable between the two groups after matching, as detailed in Table 1.

**Table 1:** Baseline characteristics of rheumatoid arthritis patients before and after propensity score matching

Characteristic	Before matching			After matching		
	RA + HCQ (*N* = 56 362)	RA + MTX (*N* = 56 362)	*P*-value	RA + HCQ (*N* = 56 362)	RA + MTX (*N* = 56 362)	*P*-value
Demographics						
Age at index (years), mean ± SD	51.8 ± 12.8	52.9 ± 12.6	<0.001	52.3 ± 12.4	52.3 ± 12.7	0.740
Female, *n* (%)	50 723 (80.0%)	49 965 (70.9%)	<0.001	44 243, 78.5%	44 357, 78.7%	0.408
Male, *n* (%)	10 517 (16.6%)	18 283 (25.9%)	<0.001	10 213, (18.1%)	10 115 (17.9%)	0.448
Ethnicity						
White, *n* (%)	38 169 (60.2%)	43 674 (62.0%)	<0.001	34 379 (61.0%)	34 405 (61.0%)	0.874
Black or African American, *n* (%)	9493 (15.0%)	9272 (13.2%)	<0.001	7788 (13.8%)	7852 (13.9%)	0.581
Hispanic or Latino, *n* (%)	5113 (8.1%)	6004 (8.5%)	0.003	4672 (8.3%)	4555 (8.1%)	0.204
Comorbidities						
Diabetes mellitus, *n* (%)	6836 (10.8%)	6997 (9.9%)	<0.001	5598 (9.9%)	5495 (9.7%)	0.303
Hypertensive diseases, *n* (%)	17 534 (27.7%)	16 485 (23.4%)	<0.001	13 738 (24.4%)	13 913 (24.7%)	0.226
Hyperlipidemia, unspecified, *n* (%)	9467 (14.9%)	9436 (13.4%)	<0.001	7607 (13.5%)	7638 (13.6%)	0.787
Chronic kidney disease, *n* (%)	3376 (5.3%)	1523 (2.2%)	<0.001	1520 (2.7%)	1486 (2.6%)	0.530
Tobacco use, *n* (%)	1519 (2.4%)	1432 (2.0%)	<0.001	1185 (2.1%)	1191 (2.1%)	0.901
Overweight and obesity, *n* (%)	8647 (13.6%)	8197 (11.6%)	<0.001	6864 (12.2%)	6884 (12.2%)	0.856
Gout, *n* (%)	1471 (2.3%)	1420 (2.0%)	<0.001	1151 (2.0%)	1132 (2.0%)	0.688
Hypothyroidism, *n* (%)	6743 (10.6%)	5455 (7.7%)	<0.001	4944 (8.8%)	4969 (8.8%)	0.793
Cerebrovascular disease, *n* (%)	2490 (3.9%)	2106 (3.0%)	<0.001	1829 (3.2%)	1818 (3.2%)	0.853
Medications						
Sulfasalazine, *n* (%)	1902 (3.0%)	2359 (3.3%)	<0.001	1710 (3.0%)	1749 (3.1%)	0.501
Leflunomide, *n*(%)	2078 (3.3%)	1404 (2.0%)	<0.001	1359 (2.4%)	1260 (2.2%)	0.050
Metoprolol, *n*(%)	5066 (8.0%)	4848 (6.9%)	<0.001	3948 (7.0%)	3964 (7.0%)	0.852
Aspirin, *n* (%)	7353 (11.6%)	7240 (10.3%)	<0.001	5822 (10.3%)	5874 (10.4%)	0.612
Atorvastatin, *n* (%)	5381 (8.5%)	6207 (8.8%)	0.039	4635 (8.2%)	4532 (8.0%)	0.262
Rosuvastatin, *n* (%)	1831 (2.9%)	1997 (2.8%)	0.547	1524 (2.7%)	1502 (2.7%)	0.685
Simvastatin, *n* (%)	1862 (2.9%)	2001 (2.8%)	0.284	1564 (2.8%)	1561 (2.8%)	0.957
Metformin, *n*(%)	3393 (5.4%)	3995 (5.7%)	0.011	3012 (5.3%)	2938 (5.2%)	0.324
Adalimumab, *n* (%)	1369 (2.2%)	2921 (4.1%)	<0.001	1340 (2.4%)	1336 (2.4%)	0.938
Golimumab, *n*(%)	183 (0.3%)	383 (0.5%)	<0.001	178 (0.3%)	202 (0.4%)	0.217
Etanercept, *n*(%)	1178 (1.9%)	2252 (3.2%)	<0.001	1134 (2.0%)	1165 (2.1%)	0.514
Infliximab, *n*(%)	250 (0.4%)	994 (1.4%)	<0.001	250 (0.4%)	285 (0.5%)	0.129
Certolizumab, *n*(%)	278 (0.4%)	397 (0.6%)	0.001	257 (0.5%)	258 (0.5%)	0.965
Tocilizumab, *n*(%)	361 (0.6%)	443 (0.6%)	0.162	317 (0.6%)	328 (0.6%)	0.664
Abatacept, *n*(%)	684 (1.1%)	731 (1.0%)	0.455	570 (1.0%)	568 (1.0%)	0.952
Tofacitinib, *n*(%)	609 (1.0%)	733 (1.0%)	0.146	519 (0.9%)	532 (0.9%)	0.687
Upadacitinib, *n*(%)	192 (0.3%)	183 (0.3%)	0.135	142 (0.3%)	159 (0.3%)	0.327

SD, standard deviation; Std. Diff., standardized difference; HCQ, hydroxychloroquine; MTX, methotrexate.

Data presented as mean ± SD or *n* (%).

The analysis evaluated the 10-year cumulative incidence of pre-specified outcomes, excluding patients with a history of the specific outcome prior to the start of the analysis window. The comparative analysis of these 10-year cumulative risks revealed statistically significant associations for several major adverse outcomes when comparing HCQ initiation to MTX initiation. Specifically, the HCQ cohort exhibited a higher 10-year RR of all-cause mortality (RR 1.379, 95% CI 1.309–1.454; *P* < 0.001) and NSTEMI (RR 1.217, 95% CI 1.103–1.344; *P* < 0.001). Furthermore, risks for pulmonary and renal complications were significantly greater in the HCQ group, including for pulmonary arterial hypertension (PAH) (RR 1.625, 95% CI 1.518–1.739; *P* < 0.001), ILD (RR 1.377, 95% CI 1.281–1.480; *P* < 0.001), and notably, ESRD, where the risk was substantially elevated (RR 2.624, 95% CI 2.267–3.037; *P* < 0.001).

In contrast, the analysis did not find statistically significant differences in the 10-year cumulative risk between the HCQ and MTX groups for other assessed outcomes. The risk of developing Alzheimer’s dementia was similar between cohorts (RR 0.907, 95% CI 0.698–1.178; *P* = 0.464). Likewise, no significant difference was observed in the risk of STEMI (RR 1.148, 95% CI 0.987–1.336; *P* = 0.074). Similarly, the risk of osteoporosis (with or without current pathological fracture) was not significantly different between the groups (RR 1.022, 95% CI 0.984–1.060; *P* = 0.258).

**Figure 2.**
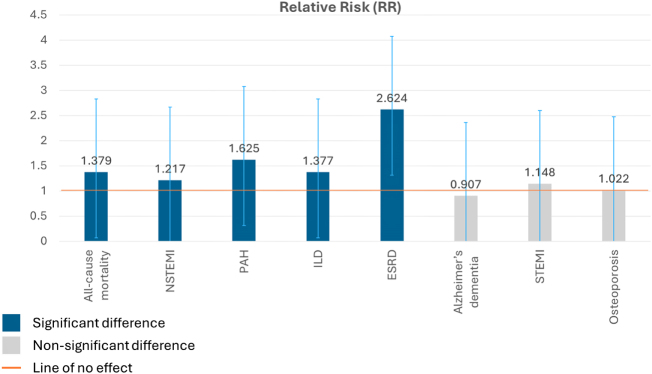
Comparative 10-year relative risks (RR) for primary outcomes in propensity score-matched cohorts.

In summary, after adjustment for measured baseline confounders using PSM, this large RWE study observed that initiation of HCQ in RA patients was associated with significantly higher 10-year RR of all-cause mortality, NSTEMI, PAH, ILD, and ESRD compared to initiation of MTX. No significant differences in 10-year RR were detected for Alzheimer’s dementia, STEMI, or osteoporosis. These results are summarized in Table 2, and a visual representation of the risk differences is provided in Fig. 2.

**Table 2:** Comparative 10-year risk of outcomes in propensity score-matched rheumatoid arthritis patients treated with HCQ versus methotrexate

Outcome	Cohort	Patients in cohort^a^	Patients with outcome (n)	Risk^b^	Risk difference (HCQ-MTX) (95% CI)	Relative risk (HCQ/MTX) (95% CI)	*P*-value^c^
All-cause mortality	RA on HCQ	56 276	3130	0.056	0.015 (0.013, 0.018)	1.379 (1.309, 1.454)	<0.001
	RA on MTX	56 302	2270	0.040			
NSTEMI	RA on HCQ	55 879	860	0.015	0.003 (0.001, 0.004)	1.217 (1.103, 1.344)	<0.001
	RA on MTX	55 924	707	0.013			
PAH	RA on HCQ	55 196	2112	0.038	0.015 (0.013, 0.017)	1.625 (1.518, 1.739)	<0.001
	RA on MTX	55 663	1311	0.024			
ILD	RA on HCQ	55 288	1703	0.031	0.008 (0.007, 0.010)	1.377 (1.281, 1.480)	<0.001
	RA on MTX	55 969	1252	0.022			
ESRD	RA on HCQ	55 992	645	0.012	0.007 (0.006, 0.008)	2.624 (2.267, 3.037)	<0.001
	RA on MTX	56 265	247	0.004			
Alzheimer’s dementia	RA on HCQ	56 315	107	0.002	−0.000 (−0.001, 0.000)	0.907 (0.698, 1.178)	0.464
	RA on MTX	56 325	118	0.002			
STEMI	RA on HCQ	56 091	358	0.006	0.001 (−0.000, 0.002)	1.148 (0.987, 1.336)	0.074
	RA on MTX	56 121	312	0.006			
Osteoporosis**ᵈ**	RA on HCQ	52 836	5079	0.096	0.002 (−0.001, 0.006)	1.022 (0.984, 1.060)	0.258
	RA on MTX	53 130	4999	0.094			

CI, confidence interval; ESRD, end-stage renal disease; HCQ, hydroxychloroquine; ILD, interstitial lung disease; MTX, methotrexate; NSTEMI, non-ST elevation myocardial infarction; PAH, pulmonary hypertension; RA, rheumatoid arthritis; STEMI, ST elevation myocardial infarction.

^a^ Patients in cohort after propensity score matching and after excluding those with the outcome prior to the 10-year analysis window. Number of patients excluded due to prior outcome for each analysis: Mortality (HCQ: 86, MTX: 60); NSTEMI (HCQ: 483, MTX: 438); PAH (HCQ: 1,166, MTX: 699); ILD (HCQ: 1,074, MTX: 393); ESRD (HCQ: 370, MTX: 97); Alzheimer’s dementia (HCQ: 47, MTX: 37); STEMI (HCQ: 271, MTX: 241); Osteoporosis (HCQ: 3,526, MTX: 3,232).

^b^ Risk = cumulative incidence at 10 years.

^c^ *P*-value for the difference in risks between cohorts (from TriNetX *z*-score output).

^d^ Includes osteoporosis with or without current pathological fracture.

## Discussion

This large-scale, retrospective cohort study leveraging the TriNetX global RWE platform investigated the comparative long-term risks of major comorbidities and mortality among RA patients initiating either MTX or HCQ^[5]^. While previous studies have compared certain risks between these agents^[2]^, our study expands on this by assessing a broader range of long-term systemic outcomes, including renal, pulmonary, and neurological endpoints, within a large, global real-world cohort. Utilizing PSM to balance baseline characteristics, our analysis revealed significant differences between the treatment groups for several critical outcomes over a 10-year period. Notably, HCQ initiation was associated with a significantly higher risk of all-cause mortality, NSTEMI, PAH, ILD, and ESRD compared to MTX initiation after matching. Conversely, no statistically significant differences were observed in the risks of Alzheimer’s dementia, STEMI, or osteoporosis based on the primary risk analysis.

It is crucial to interpret these findings within the context of established RA treatment guidelines^[3,4]^. Both the American College of Rheumatology (ACR)^[3]^ and the European Alliance of Associations for Rheumatology (EULAR)^[4]^ guidelines strongly recommend MTX as the anchor csDMARD for patients with moderate-to-high disease activity due to its established efficacy in controlling RA inflammation. HCQ is typically recommended for patients with milder disease or used in combination therapy^[1,3]^. This creates a strong potential for confounding by indication. Although PSM was used to balance measured covariates, it cannot account for unmeasured differences in underlying disease activity, which is a primary driver of treatment selection.

A central challenge in interpreting our results is the issue of selection bias. Given that MTX is generally prescribed for patients with more aggressive disease, one would expect a selection bias favoring worse outcomes in the MTX group. Our finding that the HCQ group fared worse is counterintuitive and suggests that the patient groups may differ in critical ways not captured by our matching variables. For instance, patients initiated on HCQ may have had underlying contraindications to MTX (e.g., pre-existing subclinical renal or liver issues) that also independently increased their risk for the adverse outcomes observed. Therefore, the higher risk seen in the HCQ group may reflect these unmeasured patient characteristics rather than a causal effect of the drug itself.

Our finding of higher all-cause mortality associated with HCQ initiation compared to MTX aligns with some previous research. A large US Medicare cohort study by D’Andrea *et al*^[2]^ also found an increased mortality risk with HCQ compared to MTX. This contrasts with evidence suggesting that MTX may offer cardiovascular protection^[7]^. The cardiotoxicity of HCQ is increasingly recognized, manifesting as conduction disorders and cardiomyopathy^[8,9]^, which could contribute to the observed increased risk of mortality and NSTEMI in the HCQ cohort^[8]^.

The cardiovascular findings also warrant careful consideration. The significantly higher risk of NSTEMI observed with HCQ initiation is a complex finding. While some literature suggests potential cardioprotective effects of HCQ^[1,10]^, our results align with other large observational data showing increased cardiovascular risk^[2]^. The explanation is likely multifactorial and may not be a direct drug effect. For example, residual confounding from higher disease activity or greater concomitant use of unmeasured medications like nonsteroidal anti-inflammatory drugs (NSAIDs) and corticosteroids in one group could drive this association. The lack of a significant difference in STEMI risk in our study adds complexity, suggesting potential differential effects on specific cardiovascular event types.

The associations observed for PAH, ILD, and ESRD (higher risk with HCQ) are particularly striking. While the elevated ESRD risk with HCQ is biologically unexpected based on its known mechanisms, this could be influenced by other factors. For example, chronic use of NSAIDs, which was not adjusted for, is a known cause of kidney injury and could be a significant confounder. Similarly, the higher risk of ILD and PAH in the HCQ group is contrary to what might be expected from confounding by indication, as higher RA disease activity (typically treated with MTX) is a risk factor for these conditions. This surprising finding may point to an under-recognized signal or, more likely, complex unmeasured confounding. One study has suggested that while HCQ improves immune-mediated inflammation, it can exacerbate acute kidney injury by impairing protective cellular pathways^[11]^, a hypothesis that could align with our findings but requires dedicated mechanistic study.

Finally, it is important to consider the broader context of treatment, including psychosocial factors. The therapeutic relationship and patient understanding can significantly impact outcomes in chronic diseases like RA. As discussed by Perrot *et al*^[12]^, failures in doctor-patient communication can lead to misunderstandings about treatment goals and risks. Furthermore, patient expectations and contextual factors, sometimes termed the “drucebo effect,” can influence both adherence and the perception of adverse events^[13]^. While our study could not measure these variables, they represent another layer of complexity that could indirectly influence the observed associations by affecting long-term treatment adherence and health behaviors.

This study possesses significant strengths, including its large sample size and the use of RWD. However, the findings must be interpreted considering several major limitations. First and most importantly is the high potential for residual confounding. Despite PSM, we could not adjust for key unmeasured confounders such as RA disease activity (e.g., DAS28), inflammatory markers (e.g., CRP and ESR), antibody status, functional status, corticosteroid use, or lifestyle factors, all of which heavily influence both drug choice and outcomes. Second, the study lacks data on medication dosage, treatment duration, adherence, or treatment switching. The analysis is based on initial treatment only, but patients may have discontinued or switched therapies during the 10-year follow-up, which could heavily bias the results. Third, outcomes were ascertained using ICD-10 codes without clinical validation, introducing the possibility of misclassification bias. Fourth, the analysis of numerous outcomes without correction for multiple testing increases the probability of Type I errors. Finally, no sensitivity or subgroup analyses were performed to test the robustness of the findings. Consequently, while the observed associations are noteworthy, they must be interpreted with extreme caution. These findings cannot establish causality and should be considered primarily hypothesis generating.

## Conclusion

In this large, retrospective cohort study, the initiation of HCQ for RA was associated with a higher 10-year risk of all-cause mortality, NSTEMI, PAH, ILD, and ESRD compared to the initiation of MTX. No significant differences were observed for Alzheimer’s dementia, STEMI, or osteoporosis. However, these real-world findings must be interpreted with significant caution. The observational design, inability to account for critical unmeasured confounders like disease severity, and lack of data on medication adherence are major limitations that prevent any causal interpretation. The results should be viewed as hypothesis generating and do not warrant changes to current clinical practice, which should continue to be guided by established treatment guidelines and individualized patient assessment. Future prospective studies that can capture granular clinical data and account for treatment adherence are essential to validate these findings and untangle the complex relationship between RA treatments and long-term outcomes.

## Data Availability

The data that support the findings of this study are available from the TriNetX research network, but restrictions apply to the availability of these data, which were used under license for the current study, and so are not publicly available. Data are however available from the authors upon reasonable request and with permission of TriNetX.

## Research registration unique identifying number (UIN)

The protocol for this retrospective cohort study was registered with the Research Registry (unique identifying number: researchregistry11215) and can be accessed at: https://www.researchregistry.com/register-now/#home/registrationdetails/682a13327cf8ff02d240d47b/. The study was conducted in accordance with this pre-specified protocol.

## Artificial intelligence declaration

During the preparation of this manuscript, the authors used the artificial intelligence (AI) language model Google Gemini (version Pro 2.5, accessed August 2025). The tool was used for the sole purpose of improving the language, clarity, and readability of the text. The AI was not used for data analysis, interpretation of results, or drawing scientific conclusions. All AI-generated text was critically reviewed, edited, and verified by the authors, who take full responsibility for the integrity and final content of the manuscript.

## Patient and public involvement

No patients or members of the public were involved in the design, conduct, reporting, or dissemination plans of this retrospective database study.
